# Digital Competence of Arabic-Speaking Immigrant and Refugee Older Adults Enacting Agency and Navigating Barriers: Qualitative Descriptive Study

**DOI:** 10.2196/60547

**Published:** 2025-03-25

**Authors:** Jordana Salma, Alesia Au, Ghada Sayadi, Manal Kleib

**Affiliations:** 1 Faculty of Nursing University of Alberta Edmonton, AB Canada

**Keywords:** digital competence, Arab, older adult, co-design, migrant, information and communications technology, ICT

## Abstract

**Background:**

Canada’s immigrant and refugee older adult population is projected to grow substantially, making equitable access to information and communications technologies (ICTs) vital for enhancing quality of life in older age. Strengthening the digital competence of immigrant and refugee older adults can improve their social connectedness and access to local information.

**Objective:**

This study explored the digital competence of Arabic-speaking immigrant and refugee older adults, focusing on how they engage with ICTs to meet their information and communication needs and the strategies they use to navigate digital barriers.

**Methods:**

A qualitative descriptive methodology within a social constructivist paradigm was adopted, incorporating triangulated data collection and iterative co-design cycles. The qualitative approach facilitated an in-depth exploration of participants’ experiences, skills, and emotions and the contextual factors influencing their digital competence. Data were collected through storytelling approaches, qualitative interviews, and focus group discussions, which were effective in capturing the experiential aspects of aging and technology use. Co-design cycles informed 6 digital learning sessions tailored to participants’ immediate learning needs, fostering motivation and engagement and allowing for observation of ICT use. Digital competence was mapped across the learning domains of the Digital Competence Framework for Citizens 2.2.

**Results:**

This study engaged 31 Arabic-speaking immigrant and refugee older adults residing in Canada. Most participants had limited formal education (19/31, 61%), lived with family (22/31, 70%), and reported a low income (21/31, 68%). All participants (31/31, 100%) used smartphones as their primary ICT device, whereas few (3/31, 10%) had access to a computer. In total, 3 themes were identified from the analysis, grounded in Digital Competence Framework for Citizens 2.2 competencies on information and data literacy, communication and collaboration, and safety and problem-solving. The themes focused on agency, which is enhanced or constrained using ICTs, impacting older adults’ desire and ability to use these technologies to independently meet their daily needs.

**Conclusions:**

Immigrant and refugee older adults require support to navigate digital barriers and gain digital competence. Smartphones serve as a critical tool for enhancing digital agency, which can lead to greater social connectedness and improved access to local resources in older age. The findings will inform the design of future digital competence programs for older migrants, emphasizing community partnership and reciprocal learning.

## Introduction

### Background

Information and communications technologies (ICTs) are defined as the spectrum of digital and social media used for communication and access to information [1]. Internet access rates have more than doubled for those aged ≥65 years in Canada [2]. Access to ICTs has been declared a fundamental human right, with strong evidence that the digital divide—lack of or limited access to the benefits of technology—impacts quality of life in older age [3]. ICTs provide several benefits for older adults, including social connectivity, opportunities for continuous learning, access to health-related information, and entertainment [4-8]. The benefits that older adults obtain from technology depend on the types of technologies adopted and their digital competence [9-12].

Digital competence is defined as “the confident, critical and responsible use of, and engagement with, digital technologies for learning, at work, and for participation in society. It includes information and data literacy, communication and collaboration, media literacy, digital content creation (including programming), safety (including digital well-being and competences related to cybersecurity), intellectual property related questions, problem solving and critical thinking” [13]. Digital competence is a newer concept in comparison to digital literacy, with both terms being used interchangeably [14] or viewed as different with some shared dimensions related to technological knowledge, skills, and attitudes [15,16]. Digital competence is a useful concept for older adults as it incorporates attitudes and motivations that are essential predictors of ICT use in older age [17-19]. Enhancing the benefits of using ICTs requires not only improving technological skills and knowledge but also understanding the attitudinal and socioemotional aspects of technology use [20]. Older adults are willing to adopt technology that is deemed beneficial to them and that has positive implications for their daily life [6,21,22]. Older adults use ICTs to maintain social connections, strengthen family ties, and support activities of daily living and leisure [22,23]. These digital tools can enhance older adults’ independence, autonomy, and self-confidence [12,22]; however, the digital divide persists due to a range of factors, such as living with disabilities, lack of social support, lower educational level, and low income [12,24]. During the COVID-19 pandemic, physical distancing and the health risks to older adults necessitated creative digital solutions, such as ICT-mediated social and recreation programs, which reinforced the potential benefits of ICTs [25,26].

Improving digital access and competence is one strategy to support healthy aging in migrant populations. Canada continues to welcome high numbers of immigrants and refugees, with an increasing percentage from Arabic-speaking countries [27]. Younger cohorts of immigrants demonstrate high levels of proficiency in using technologies to adapt within postmigration contexts [28-30]. The extent to which this proficiency applies to immigrant and refugee older adults has been less explored despite well-documented ICT adoption to facilitate social engagement and sustain family ties [31,32]. Older Arabic-speaking migrants experience loneliness, social isolation, and barriers to access and use of aging-supportive services [33,34]. Arab migrants traditionally belong to extended family models and value close interpersonal relationships within local and transnational kin networks [35,36], with the adoption of ICTs influenced by social identities and roles [37,38]. In addition, gender, income, educational level, and technology infrastructure in their countries of origin shape the use of technologies and motivations to develop particular knowledge and skills [24,39,40].

To support digital competence and create avenues for digital learning, it is critical to understand the individual and social influences on ICT use among older migrants [41]. The lack of theorization linking aging and technology use is well cited in the literature [42], with little integration of immigrant and refugee older adult perspectives in gerontechnology [37]. This study aimed to explore the “messiness of practice” [43] that sheds light on the ways in which immigrant and refugee older adults interact with technologies in their daily lives to solve problems, their exercise of agency, and their digital competence.

### Objectives

This study aimed to explore the digital competence of immigrant and refugee older adults with a specific focus on (1) the ways in which immigrant and refugee older adults engage with ICTs to meet their needs for accessing information and communication and (2) the strategies used to problem solve and navigate barriers to using ICTs.

### Conceptual Framework

The Digital Competence Framework for Citizens (DigComp) 2.2 encompasses 5 key areas defining the scope of digital competence: information and data literacy, communication and collaboration, digital content creation, safety, and problem-solving [44]. The initial 3 domains focus on competencies associated with particular tasks and applications. The fourth and fifth domains, “safety” and “problem-solving,” are relevant across all digital activities. Each competence area incorporates skills, knowledge, and attitudes that are essential for digitally literate citizens, with proficiencies that range from basic to advanced and that can be used to design competence assessment tools, support education, and tailor training [45]. This framework was selected for its comprehensiveness and its attention to attitudes across the range of competence areas that were deemed relevant to older adults in a growing technology-dependent world [5,46,47].

## Methods

### Research Design

A qualitative descriptive methodology within a social constructivist paradigm was used and included triangulated data collection methods and iterative co-design cycles with immigrant and refugee older adults to explore ICT use, digital competence, and related learning needs. A qualitative approach was selected to allow for in-depth experiential explorations of digital competencies. The goal was to conduct a holistic investigation into the ways in which older adults engaged with technologies, including their skills, knowledges, emotions, and influencing contextual factors. Triangulation of data sources over a period is required to obtain descriptively rich insights on technology use in older age [48]. In addition, older adults respond best to storytelling approaches for data collection that are flexible and engaging, which makes qualitative interviews and focus group (FG) discussions most effective in expanding understandings of aging [49]. Co-design cycles informed a sequence of 6 learning sessions to address immediate learning needs identified over the course of data collection as older adults’ motivation and interests are key drivers of digital engagement [12,20]. Reciprocity via supporting their immediate digital learning needs helped meet ethical obligations to the community and build sustainable partnerships [50].

### Ethical Considerations

This study received ethics approval from the University of Alberta Research Ethics Board (ethics number Pro00103587). All participants provided informed consent before data collection and were informed of their right to withdraw at any time during the study. Identifying information was removed from interview and FG transcripts and from observation notes and then shared with the research team for analysis and knowledge dissemination. Participants were provided with an honorarium of CAD $50 (US $35.20) for participation in the data collection sessions.

### Sample and Recruitment

Study participants were community-dwelling individuals who (1) were aged ≥55 years, (2) self-identified as first-generation Arabic-speaking migrants, and (3) reported using ICTs in their daily lives. We selected the age of ≥55 years to define “older adult” because some refugees in the Arab community identified as older adults at <65 years [51,52]. Despite the World Health Organization using the age of ≥60 years to define older adults, the definition acknowledges variations in life expectancy and cultural perceptions of aging worldwide [53]. Cohorts of Arab-Canadian migrants differ in levels of education, reasons for migration, religious traditions, and experiences of macro and micro historical and political events [54]. Most Arab older adults in this study were Syrian, Lebanese, Jordanian, or Palestinian; ascribed to the Muslim faith; and had varying levels of education, income levels, and English-language fluency. A social service organization that catered to newcomers and a mosque that included a congregation of Arabic-speaking older adults supported the project by hosting the data collection sessions and aiding recruitment. Flyers in English and Arabic were distributed by the 2 partner organizations, and staff helped explain the purpose of the study to potential participants. These organizations also provided space for the group-based digital learning sessions. Purposive sampling began with service providers at these organizations connecting with immigrant and refugee older adults via phone calls and in person to introduce the study. Immigrant and refugee older adults with interest in participating were then contacted by the research team for screening and formal consent to participate. Data saturation was determined by repetitive patterns of experiences, perceptions, and needs identified in the data over the course of the study.

### Data Collection

#### Overview

Data collection included 2 phases. Phase 1 involved individual (n=8) and group (n=2) semistructured interviews to explore experiences of using ICTs. Phase 2 included digital learning sessions where observations and FGs facilitated a detailed documentation of immigrant and refugee older adults’ digital competencies and learning needs. Triangulation of data sources over a period allows for access to descriptively rich insights on technology use in older age [22,38]. All individual interviews and FGs were audiotaped and transcribed verbatim by a professional transcriptionist trained in the Arabic language.

#### Semistructured Interviews

**Figure 1:**
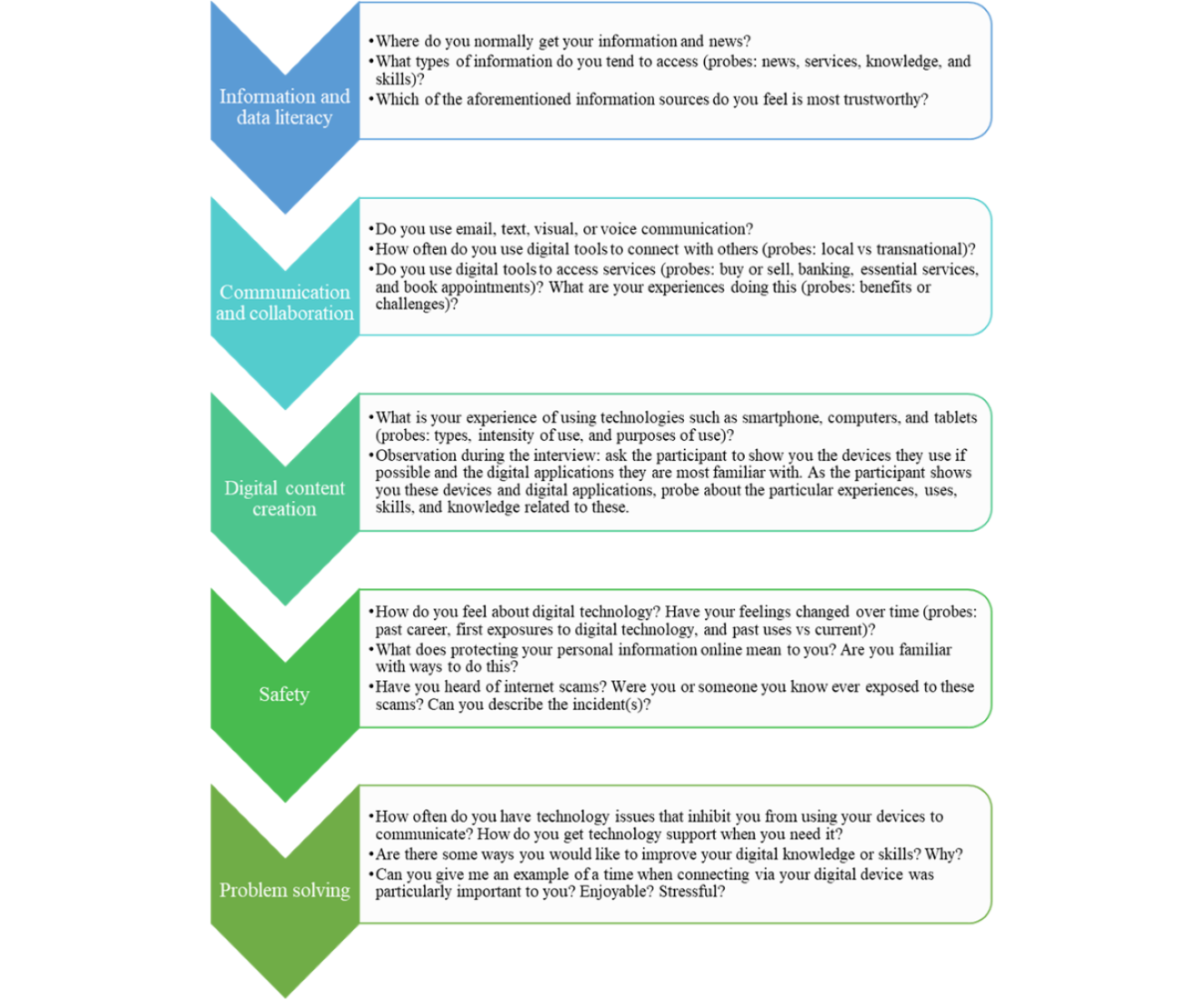
Phase 1 interview questions from the Digital Competence Framework for Citizens 2.2.

Individual interviews were conducted in participants’ homes from April 2022 to June 2022 by an Arabic-speaking interviewer and lasted, on average, 1 to 1.5 hours. For the first phase, an interview guide using the DigComp 2.2 guided the types of questions asked (Figure 1). Learning session topics were derived from the priority areas identified in the phase-1 interviews and included learning about the essentials of smartphone hardware and software, social media communication tools such as Facebook and WhatsApp, and using email and online access to information.

#### Co-Design Sessions

**Figure 2:**
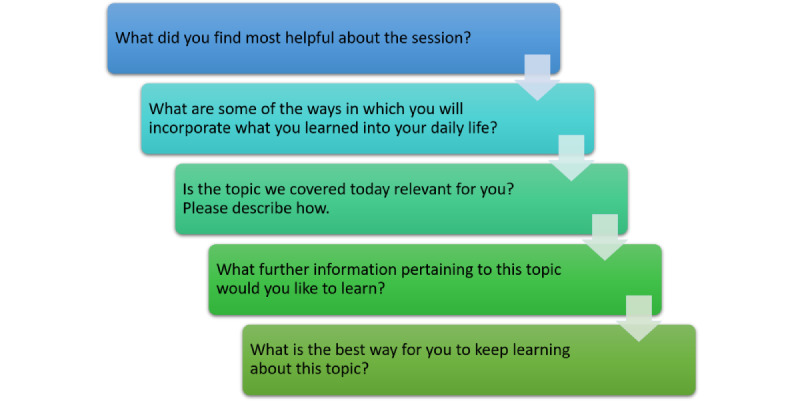
Phase 2 focus group guiding questions.

In phase 2, we used a participatory framework [12,20] to co-design ICT learning sessions between September 2022 and November 2022. The session instructor was a computer engineering university student, and he was supported by an Arabic-speaking research assistant who translated for participants who only spoke Arabic. The participatory co-design framework facilitated collaborative approaches that actively involved older adults in the design and decision-making processes of the learning sessions to ensure that the sessions were engaging, built on the lived experiences of participants, and met their self-identified learning needs [12,20] (Au et al [55] reported in detail on the co-design process). The project lead, who mentored the session instructor and research assistant, had >10 years of experience in participatory research methods and provided training on co-design processes during weekly team meetings. In each session, participants started with a 30- to 45-minute FG discussion related to the main learning topic of the day to identify the ways in which they engaged with that particular ICT in their daily lives; the challenges they faced; and their attitudes, emotions, and learning needs (Figure 2). Phase 2 FGs were used to deepen insights from phase 1, triangulate data for validation, tailor learning sessions to participants’ needs, and engage them in identifying their learning requirements and experiences with digital technologies. Feedback on each learning session helped the instructor refine subsequent session delivery and content. The purpose of this study was not to develop or test a complete digital learning program; rather, the purpose was to create a supportive environment where immigrant and refugee older adults had some direct benefit from participating in the research study while the research team gained in-depth understanding of their digital practices. Participants for these sessions were divided into 3 groups, with separate men’s (3/21, 14%) and women’s (10/21, 48%) groups in the faith-based organization and a mixed-gender group (8/21, 38%) in the social service organization. Observations (54 hours of observational data) that occurred during these sessions entailed a researcher documenting group learning processes and the digital competencies of participants as they navigated their devices and worked together through lesson material.

### Data Analysis

**Figure 3:**
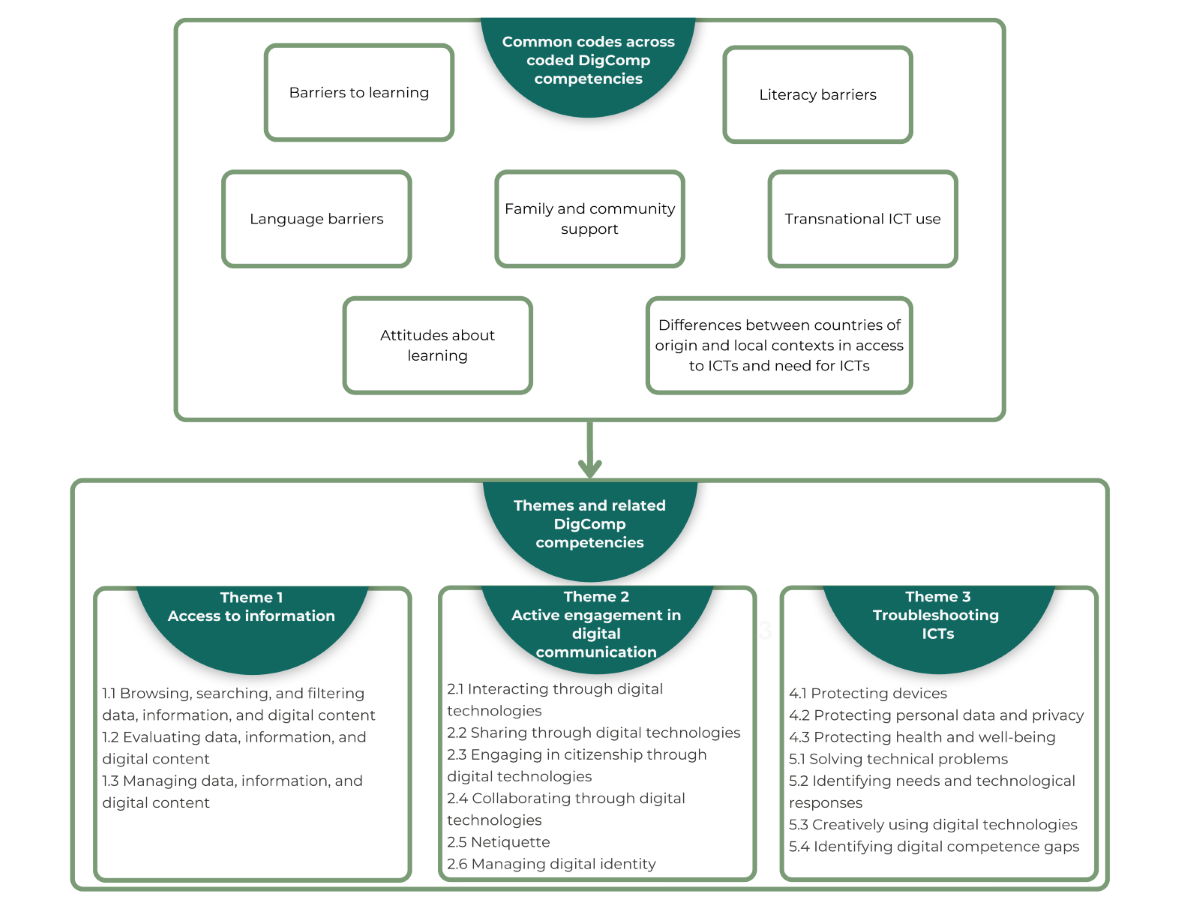
Digital competencies and thematic analysis process. DigComp: Digital Competence Framework for Citizens; ICT: information and communications technology.

Arabic conversations were initially analyzed by bilingual team members, with key quotes and codes then translated into English for team discussion and use in knowledge dissemination. A reflexive thematic data analysis approach was used to identify, analyze, and report patterns within the data [56] facilitated by the NVivo qualitative data management software (QSR International). The 6-phase approach included familiarizing ourselves with the data, generating initial codes, searching for themes, reviewing themes, defining and naming themes, and writing a report [57]. Initial codes were organized around the DigComp 2.2 competencies, followed by linking and comparing codes within and across competencies to arrive at overall patterns of understanding about participants’ experiences of engaging with and learning about ICTs (Figure 3).

To ensure trustworthiness in data analysis, we used peer debriefing, data triangulation, and reflexivity [58]. Peer debriefing provides external validation, which was done through weekly team conversations to discuss the ways in which observation, FG, and interview data reflected particular competencies. Data were analyzed iteratively, meaning that the data were coded systematically once collected and then recoded as codes became more refined over time with deeper understanding. Team consensus was reached with a topical coding tree based on the DigComp 2.2 competencies. One team member (JS) led the refinement of coding to develop the final themes for this paper, and all coauthors provided critical feedback at multiple points during this process. The authors have expertise in qualitative methods, aging, and migration. In total, 2 authors are bilingual Arabic-speaking academics, with the remaining authors being second-generation or first-generation immigrants to Canada and academic researchers.

## Results

### Overview

A total of 31 immigrant and refugee older adults participated in this study, with an average age of 64.1 (SD 7.7) years. Most participants were women (20/31, 65%), identified as Arab (29/31, 93%), and were immigrants (19/31, 61%) or refugees (10/31, 32%). The highest level of education was primary or secondary school for most participants (19/31, 61%). In total, 2 non–Arabic-speaking participants were included because they were spouses of Arabic-speaking participants attending the program. More than half of the participants lived with their spouse, their children, or both (22/31, 71%); reported a family income of <CAD $20,000 (US $14,078.30; 21/31, 68%), which is categorized as low income in Canada; and received some form of government financial support (21/31,68%; Table 1).

All study participants (31/31, 100%) used smartphones as their primary ICTs, with only a few (3/31, 10%) reporting having access to and knowing how to use computers. Smartphones were cost-effective, accessible, and familiar to immigrant and refugee older adults, and hence, the focus of the learning sessions throughout this study was on exploring ICT use through these devices. The themes highlight the ways in which agency is enhanced or constrained using ICTs and draw attention to older adults’ desire to use these technologies independently to meet their daily needs. The 3 themes were developed from across four dimensions of the DigComp 2.2: (1) information and data literacy, (2) communication and collaboration, (3) safety, and (4) problem-solving. The dimension of “digital content creation” was not inductively discussed by participants, and the scope was beyond the capacity of this study to address (Multimedia Appendix 1).

**Table 1:** Demographic characteristics of the participants (N=31).

Characteristics			Overall		Phase 1 (n=10)		Phase 2		
							Organization 1 (n=9)		Organization 2 (n=12)
Age (y), mean (SD)			64.1 (7.7)		61.2 (3.1)		64.2 (6.0)		66.3 (9.5)
**Gender, n (%)**									
	Women	20 (64)		5 (50)		5 (56)		10 (83)	
	Men	11 (36)		5 (50)		4 (44)		2 (17)	
**Country of birth, n (%)**									
	Lebanon	5 (16)		0 (0)		1 (11)		4 (33)	
	Syria	16 (52)		5 (50)		6 (67)		5 (42)	
	Palestine	3 (10)		1 (10)		0 (0)		2 (17)	
	Sudan	3 (10)		3 (30)		0 (0)		0 (0)	
	Egypt	2 (6)		0 (0)		1 (11)		1 (8)	
	Afghanistan	1 (3)		0 (0)		1 (11)		0 (0)	
	Iraq	1 (3)		1 (10)		0 (0)		0 (0)	
**Dwelling, n (%)**									
	Spouse	10 (32)		1 (10)		6 (67)		3 (25)	
	Children	2 (6)		1 (10)		1 (11)		0 (0)	
	Spouse and children	10 (32)		5 (50)		0 (0)		5 (42)	
	Alone	6 (19)		1 (10)		1 (11)		4 (33)	
	Other	3 (10)		2 (20)		1 (11)		0 (0)	
**Family income, n (%)**									
	<CAD $20,000 (US $14,078.30)	21 (68)		7 (70)		7 (78)		7 (58)	
	CAD $20,000 to CAD $40,000 (US $14,078.30 to US $28,156.60)	7 (23)		1 (10)		2 (22)		4 (33)	
	CAD $40,000 to CAD $60,000 (US $28,156.60 to US $42,235)	2 (6)		1 (10)		0 (0)		1 (8)	
	Unknown	1 (3)		1 (10)		0 (0)		0 (0)	
**Time lived in Canada (y), n (%)**									
	<5	12 (39)		5 (50)		2 (22)		5 (42)	
	5-10	8 (26)		3 (30)		4 (44)		1 (8)	
	>10	11 (36)		2 (20)		3 (33)		6 (50)	
**English or French fluency, n (%)**									
	Excellent	3 (10)		2 (20)		1 (11)		0 (0)	
	Good	13 (42)		3 (30)		4 (44)		6 (50)	
	Minimal	14 (45)		5 (50)		4 (44)		5 (42)	
	None	1 (3)		0 (0)		0 (0)		1 (8)	
**Highest educational level, n (%)**									
	Primary school	9 (29)		1 (10)		2 (22)		6 (50)	
	Secondary school	10 (32)		2 (20)		4 (44)		4 (33)	
	Postsecondary school	11 (36)		7 (70)		2 (22)		2 (17)	
	Other	1 (3)		0 (0)		1 (11)		0 (0)	

### Theme 1: “Here You Need to Go on the Website and See for Yourself” (Access to Information)

All participants described using their smartphones to access information via social media platforms, with WhatsApp and Facebook being the most used, followed by YouTube. Search engines such as Google were rarely described as information sources by participants. All participants were skeptical about the credibility and authenticity of the information on social media. Participants most often used family and friends for information checks to confirm or disconfirm what they viewed on these platforms:

Yes, I subscribed with (news media channel) two or three times but I’ve seen fake news on there because we hear things and they broadcast different and fake things. We ask our friends and family in Iraq if the stuff they talked about has really happened there and they say “no,” so all their news is fake.

P5; older man; interview

Immigrant and refugee older adults were savvy in evaluating information, especially with regard to world events. The information accessed related to Canada and their countries of origin as they followed ongoing political and social events. Accessing trustworthy information was heavily reliant on connections within transnational social networks that were made possible via the use of ICTs to communicate at a distance via free social media apps that allowed for texting, voice messages, and phone calls to gather information in real time:

Nowadays, to get the right information, sometimes I have to check more than one source and I compare the information...I exchange information with my friends and see if it’s accurate. See what they’ve heard, so they tell me just to try to get the most accurate information...some [friends] are here, and some are back home. It depends. Some in Egypt, some in a different country.

P12; older man; interview

Beyond social and political news, participants used ICTs to search for information related to their hobbies and religious practices and to resources for immigration and health information:

For entertainment on YouTube I go and see how to bake a cake, I tried banana bread, amazing. I make things if I like the recipes.

P5; older woman; FG

Participants often received links to videos and images on social media that allowed them to explore a range of topics of interest, and many reported high daily uses for entertainment and information access:

Sometimes you will find it [health information] on YouTube and sometimes on Facebook. I type the name of the doctor on YouTube and I listen to what he says about health and about medicinal herbs...if I think the information is correct I follow it and if I do not think it is correct I do not follow it.

P1; older woman; FG

Refugee newcomers struggled as they quickly realized that they were expected to access online information needed for settlement. These participants were socially isolated during the COVID-19 pandemic and struggled to use email to access information critical to their daily lives, such as opening newsletters, paying utility bills, reading and responding to government emails, and accessing online banking:

No, so they don’t know anything about accessing health information or banking information online, or any essential services, my understanding.

Translator for P1 and P2; older couple; group interview

While newcomers are connected to a settlement worker in Canada for their first year, this support was not always consistent or available in a timely manner or did not continue for a sufficient length of time:

Even here when you go to a government office to do anything, for example, they give you a website and ask you to see for yourself...in Syria, they would do that for you, if you needed anything, government employees would communicate clear instructions and you would get it done. Here you need to go on the website and see for yourself and this is hard for seniors, other than the language problem, they don’t even know how to open or go on a website. For example, even though I am able to rely on myself, I still find it difficult because of the language. Even when I go on certain websites, their website design is sometimes a little different in comparison to what I was used to.

P12; older man; interview

All participants spoke Arabic, Kurdish, or Farsi as their native language, displaying diverse levels of proficiency in both written and spoken English. Language posed a challenge to browsing, searching, and filtering digital content in English. Consuming information via social media and specifically audiovisuals was easier for participants. Accessing information via ICTs was challenging not only due to lower digital competence and language barriers but also due to skepticism about the trustworthiness of the information and the applicability of this information to their unique situations. Furthermore, newcomers struggled with engaging with ICTs after migration due to the difference between technology in Canada and in their home country as they had shifted into a digital world later in life driven by the necessities of migration.

Finding trustworthy sources of information can be difficult for isolated immigrant and refugee older adults who do not have strong local social networks. Language barriers prevented accessing local trusted sources of information, such as health care practitioners and service providers:

There are issues with accessing banking or health information because often we opted to ask people around us locally, but have found that the information that we gathered could be inaccurate and the wrong information can spread quickly.

P6 and P7; older couple; group interview

Finding these trusted individuals was especially challenging for newcomers who might not know how to find a physician who speaks their language or might be unaware of older adult service providers in their local region. Arabic social media groups and social media influencers who shared information after migration about settlement in Arabic were especially helpful:

In relation to the YouTube English lessons, someone who used to be an engineer in Syria, he started making YouTube videos in Edmonton that is English and Syrian translation. This is the content that they watch and he get the video link through WhatsApp through his cousin. They shared that thanks to the YouTube algorithm, they can watch the rest of his videos on his channel.

P7; older man; interview

Immigrant and refugee older adults who had English-language fluency or strong social connections within local Arabic-speaking communities were better connected to needed information and did not report these challenges. These participants reported more nuanced challenges such as storing and retrieving information:

I don’t know how to [retrieve and save information that I have accessed]...I don’t know where to save it.

P2; older man; FG

Others struggled with understanding search algorithms and the ways in which data were filtered by social media platforms, which caused frustration and inhibited access to preferred information:

P3 has a question about how to cancel certain posts that she sees on Facebook. She is confused as to why they show up when she has never pressed “like” on these posts.

Older woman; observation

In summary, immigrant and refugee older adults described a high level of skepticism about the information they found on social media, but often this was the most accessible source of information for those who were isolated with low digital competence and language barriers. Immigrant and refugee older adults were more comfortable navigating social media platforms in Arabic, and the availability of voice and video options supported those with low reading literacy. This meant that immigrant and refugee older adults were subjected to social media trends and algorithms that might bias what information they saw. This was countered by a reliance on trusted social contacts locally and transnationally to verify information from these digital sources. This coping strategy was ineffective in local contexts, where newcomers with few social contacts lacked access to reliable and needed information about their health and aging-supportive resources after migration.

### Theme 2: “We Are Mute” (Active Engagement in Digital Communication)

A variety of platforms were used to meet participants’ needs to communicate with family, friends, and others in their social networks. The most common social media platforms used were WhatsApp and Facebook. For participants who were illiterate or had low literacy, the voice message and video features of these platforms allowed them to engage with others. Some individuals used Zoom for group lessons and meetings with family. Email was the least used communication tool. Having left Syria before electricity and the internet were widely available in their village, one participant explained how the need and opportunity to use social media to connect with family began with their migration journey to Turkey—“We didn’t know Facebook in Syria not even WhatsApp...when we went to Turkey we started a little to learn” (P1; older man; FG)—whereas the use of email followed to communicate with immigration officials and service providers in Canada. Responding to emails can be particularly important to access government social and health services, but a combination of language barriers and digital competence struggles prevented some newcomer refugees from using this communication tool:

P1 after seeing this slide has mentioned that she gets email messages and she doesn’t understand, she doesn’t open the message and it is left unread. Doesn’t know how to respond to voicemail.

Older woman; observation

Participant knows how to open Arabic emails and uses it to track for school or government, doesn’t send emails himself due to English barrier, and he communicates through social media more.

Older man; observation

Agency to create a digital identity and manage communication with others was important for participants. Some participants only received communication and were not able to reciprocate or initiate communication, which was a source of frustration:

I have been here 46 years, I went to school, I went to high school, I went to university, I did my education. At the time, I did all those but no technology was at the time, and now I have been retired for 10 years. Like on Facebook, if somebody sends me a message, I go back and answer them but for me to open my telephone and call them or send them a message, I do not know how.

P8; older woman; interview

Similar comments were made by women in the study, who reported wanting to learn to use email to expand beyond the usual familiar tools:

To enter into society...meaning if my friend sends me an email, I want to learn to respond to her...we read the message but we don’t know how to respond.

P6; older woman; FG

Other participants reported communicating by proxy, where family members navigated their devices for them, responding to emails for communication with service providers and initiating contact with family and friends:

Sometimes there are things like the skype and internet-related things like video calls when I talk with my kids, my wife helps me with that. I am not very good with technology because we’re a bit old school.

P10; older man; interview

While this support was appreciated by some, most reported wanting independence to alleviate the burden on their children. There were frequent comments about frustration with not keeping up with the times, being behind their grandkids in understanding ICTs, and not being able to maximize the use of their devices:

...but how much help from your kids! You know they are always busy, especially for mama, ok later later, tomorrow, tomorrow, and then tomorrow they never showed up for me.

P8; older woman; FG

For most, the lack of agency was revealed in observations during learning sessions despite their assertion initially that ICTs were accessible and had a high degree of integration into their daily lives. Immigrant and refugee older adults had difficulty creating digital content, such as initiating picture sharing, starting conversations, and deleting posts. Most participants struggled with these skills and required assistance to learn how to complete them during the learning sessions:

P1 knows how to share existing posts, is unable to save her own picture and share it.

Observation

This resulted in engagement being one-sided, eliminating the participants’ autonomy to connect on their own terms to support preferences and needs. Passive use of ICTs, where participants observed but did not comment or create digital content, was common:

I access Facebook daily. I do not participate much but I benefit from world news, political news, religious news.

P8; older woman; FG

A lot of the participants don’t post, rather they are looking at posts.

Observation

For those who had basic competence levels to communicate by posting, sending voice or text-based messages, and responding to social media posts, the more technical features of these applications were cited as challenging, such as deleting posts, adding friends, hiding posts, and creating private groups. Learning to block others, create private groups for discussion, and add or delete contacts is essential for agency in digital spaces:

P3 mentioned that she only blocks people on her phone, not on Facebook. She thinks it is really important to learn how to do this. Going over the Facebook slide, P4 wants to learn more about it...She knows how to add people and to search for content. She doesn’t know how to block people on Facebook.

Observation

Transnational communication was prevalent within this population, and many participants connected with family, friends, and acquaintances overseas, with the lack of digital skills translating to some evidence of gendered power inequities, where women reported not being able to filter information or restrict access by families within transnational spaces:

P5 has a large friend request list containing her in-law side of the family. Although P5 knows them, she didn’t want to add them as her friends on Facebook. She wants to keep her privacy, and noted that her husband has a large family (largest family in town), and she doesn’t want them on her Facebook. But G. noticed that she also doesn’t really post. H. wonders if once we teach them how to manage privacy settings on posts, maybe she may want to post more and may feel more comfortable to do so.

Observation

Communication via ICTs was an ongoing feature of participants’ lives, and for some who were physically more isolated, it was the main source of daily communication. While, on the surface, it appeared that immigrant and refugee older adults had a high degree of competence due to their intense reliance on ICTs, it became apparent that agency was constrained due to limited control over how, when, and what was communicated. Creating digital content, communicating with preferred social contacts, and using communication to access local sources of support were major challenges.

### Theme 3: “Don’t Give Me a Fish, but Teach Me How to Catch It” (Troubleshooting ICTs)

Safety online and troubleshooting to address issues such as storage, function changes, and device accessibility were challenges for immigrant and refugee older adults. Safety related to sharing information online, password management, and privacy in online communication. Participants relied on practical approaches to store their passwords, such as digital word documents, notebooks, or family members. Learning sessions were often interrupted to troubleshoot password and security functions of their devices:

Their bewilderment and lack of awareness says a lot—they’re learning that their children have set up a lot of the functions on their phones for them...so the participants never needed to deal with the set-up process and account/password management.

Observation

One participant described using the Notes app on their phone to store their passwords but was fearful as to what would happen to their digital identity if they misplaced their phone:

She has issues with remembering her passwords. She had written down her passwords in her phone but when she lost her phone, she couldn’t recover her passwords.

P6; older woman; FG

Participants had a difficult time creating strong passwords and tended to use the same password across applications. They reported trouble remembering passwords and were required to use the password reset feature, which posed additional complexities. One participant felt overwhelmed with the number of passwords that are required to use ICTs and the need to synthesize complex passwords:

Yeah, I can’t deal with, you know, like if I want to search about something on a Google, I pray, I go, or if they ask me to put my password, probably I have 200 passwords and I don’t know which one. You know, like I’m not capable to action and I write down my password. I write down my email, whatever, and the next day I forgot something or some letter.

P10; older man; interview

Generally, the participants were well equipped to deal with security issues. There were instances in which participants could recognize scam calls and phishing emails. They were hesitant about making new friends online, joining Facebook groups that they did not recognize, and sharing photographs online. This hesitance was outlined by one participant:

You don’t trust like on the Internet to find somebody new, or you don’t know nothing, and you don’t know if he’s a con artist or thieves or, you know, eat your life. I don’t like to meet those people that I don’t know them. But if I knew a neighbour, you know, “Good morning, good afternoon.” She visits me, I visit her. Yeah, I would like to communicate and be a friend and help her and she help me, accepted. But somebody like introduced, met on a—or I met on the Internet, and I don’t believe those to be follow.

Older woman; FG

Instances of scams were not described by participants except for one who talked about learning to avoid accepting phone calls from unknown numbers. While most participants reported not responding to calls from individuals whom they did not know, this participant described in detail learning to be safer using ICTs after falling victim to a scam:

In the beginning, I have many problems with this. Sometimes I answer and I give my, you know, my information. Yeah, because, when I came here, I thought that it’s trustworthy...this is a country of laws and never imagined this type of stuff would happen here to be honest. I had a few issues and I even had to cancel my credit card and so I learned, so now, whatever I don’t understand, I don’t engage in...Sometimes I don’t even answer and I miss appointments... Sometimes they are calls from Canada that are important for me and I miss them.

P12; older woman; interview

Privacy when using digital devices was of concern for most participants, who tried to navigate this by not sharing or posting on social media and not adding people who were not part of their social network:

Daily I get friend requests...people I don’t know so I ignore them completely.

P3; older woman; FG

Privacy for women related to religious and cultural expectations of modesty and to perceptions of avoiding envy or gossip. Simply not posting was easier versus restricting privacy settings so only certain people could view the posts. According to one participant, “I do not post my pictures on Facebook” (P4; older woman; FG). Another participant agreed and added that “because this is something private, it is not necessary for the whole world to know about it” (P3; older woman; FG). A group of women discussed an incident in which a video of their social gathering, involving clapping and singing, was shared without the consent of one of the participants on social media. Due to religious views about modesty, the women requested that this video be removed. The conversation centered on the importance of privacy and consent when posting content on public forums:

It was a misunderstanding...we let our guard down with our friends...she was afraid and didn’t want this to spread.

P4; older woman; FG 3

However, often, immigrant and refugee older adults were unaware of how to navigate privacy settings on different devices and platforms:

Activity on how to make account more private: Participants needed to be verbally and individually guided to find the settings menu for privacy. All participants changed their settings to be more private for friends’ requests, even when facilitators described it in a neutral way.

Observation

Troubleshooting for accessibility of devices was an issue in that not everyone knew the possibilities with Google Translate and other apps. Many participants were only literate in their first language, Arabic, and some were illiterate in Arabic, so they used their phones to watch and listen but not to read:

The message comes to us via phone and we understand a little of it but we then take it to someone who reads English, a friend...I send it to him via phone and he tell me what it is about.

Older man; FG

The use of text-to-voice apps and translation functions was not described by most participants outside of receiving and sending voice messages using social media platforms. Most participants who had family living with them or nearby used them for support with troubleshooting their devices. Immigrant and refugee older adults all preferred to learn how to troubleshoot on their own without relying on family and wanted community supports to help expand their digital competence. One participant shared that “They don’t have time” (P6; older woman; FG). Another participant agreed and shared that family members lacked patience as “They take the device from you, do this and that, okay, how did you do that? How will you learn it if you are not doing it? Don’t give me a fish, but teach me how to catch it” (P1; older woman; FG).

Some immigrant and refugee older adults did not have family or friends nearby or lived alone:

I don’t have somebody live with me, so when I stuck, I get so upset so that’s why I never try again.

P4; older woman; interview

These individuals reported frustration and a desire to access support to enhance their digital competence, citing this as a main reason for joining the digital learning sessions. Participants rarely reported accessing services or programs that help with digital competence, especially those who were newcomers to Canada and felt unfamiliar with avenues for this type of support. None reported using the library or other mainstream technology support programs for older adults. Local ethnocultural and faith-based organizations and acquaintances were cited as helping at times:

...there is no one to help us, we do not have children here, they are all in Syria and Jordan and Lebanon...We take it to the mosque or to someone we know.

Older men and women; FG

Overall, immigrant and refugee older adults experienced significant challenges navigating the technical features of their devices, which, in turn, restricted the scope and range of digital engagement. Assistance from family, when available, was often a source of support to use ICTs. Immigrant and refugee older adults preferred to expand their knowledge and skills to gain further independence in navigating their devices. Participants did not report enrolling in any formal digital learning programs before taking part in this research project.

## Discussion

### Principal Findings

This study explored the ways in which immigrant and refugee older adults use ICTs in their daily lives, their sense of agency, and their digital competence. This study addressed a significant gap by focusing on digital competence among Arabic-speaking older adults, a population largely overlooked in existing research. While the DigComp 2.2 framework has seen limited application with older adults, its detailed competency structure offers valuable insights into learning needs and serves as a robust tool for future research and interventions. The findings highlight how personal digital competence influences older migrants’ engagement with ICTs for local and transnational purposes, providing actionable insights for designing interventions or exploring specific competencies. In addition, digital agency emerged as a critical yet understudied construct, particularly within the context of aging and migration, warranting further exploration.

The sociotechnical dimensions of ICT are described as the broader social practices and structures within which immigrant and refugee older adults are embedded and which shape their ICT use and choices [59,60]. Participants primarily relied on social media platforms to access information, entertainment, and social connections. Similarly to other migrant populations, Arabic-speaking older adults use a range of technologies to maintain transnational social networks, cultivate belonging to places of origin, practice valued social roles such as grandparenting, and navigate barriers to local integration [38,61,62]. Observing older adults use ICTs during the learning sessions was a rich data source, highlighting their creativity and agency despite constraints imposed by low digital competence.

Agency relates to using ICTs in ways that meets one’s preferences and needs [5]. Agency has been a core concept in understanding the interplay of internal and external factors that shape technology adoption and the impacts of technology use [60]. Participants in this study struggled to meet their needs via ICTs and, simultaneously, exercised agency to overcome digital barriers. Digital use was on a spectrum from low to advanced agency in managing digital tools to meet needs and preferences during and after settlement in Canada. Constrained agency was most evident when lower digital competence was coupled with minimal environmental and social support to engage with ICTs. This was demonstrated when participants could not search for needed information on online platforms, were unable to control who they communicated with via blocking a contact or creating a private friend group, and stopped using particular apps or phone features because they did not know how to troubleshoot their devices. While immigrant and refugee older adults lacked individual digital competence (microlevel barrier), they were also disadvantaged by technosocial systems that were not designed with and for older migrants (system-level barrier).

Digital competence in searching for and filtering information was limited, especially when seeking information related to settlement or aging-supportive resources. This study showcases multiple exemplars of unsupportive technosocial systems for older migrants. Information about services for older adults is increasingly digitized, which makes it inaccessible to those with poorer digital and other literacies. A mismatch was evident between what services were available and what immigrant and refugee older adults had access to, which is mirrored in other studies on service access barriers [37]. Arabic translations of social and health service information are sparse in Canada despite the Arabic-speaking demographic increasing steadily over time. While immigrant and refugee older adults preferred information shared through trusted social networks, either in person or ICT mediated, newcomers had limited access to this avenue after migration. This was especially evident for refugee newcomers who had arrived during the pandemic, which mirrors what has been reported elsewhere [63,64]. While Arabic social media platforms are readily available, participants felt that the information was not always trustworthy or tailored to their local needs. Technologies, even when not adopted by older adults, influence their lives by changing the social world around them [59]. Study participants were deprived of local knowledge due to limited access to government websites and difficulty searching for online information in English, whereas the Arabic social media platforms that they did use were not specifically oriented to older adults living in Canada. The example of an Arabic-speaking social media influencer who provided local knowledge of the city showcases the potential for social media as a place for effective knowledge dissemination to older migrants.

Despite constrained agency, immigrant and refugee older adults were not always passive ICT users but rather adapted to, overcame, or disengaged from these technologies [59]. Participants disengaged from some ICT-mediated activities such as sharing content on social media because of privacy concerns and their lack of competence in controlling privacy settings or creating private groups. Participants used creative ways to store information, such as sending the information to a peer via WhatsApp so that it was stored in the conversation thread. “Hybrid practices,” where technology is used to adapt ways of doing things [5], were also evident, such as accessing information via social media and then cross-checking accuracy with a trusted social network. Stereotypes of older adults influence the technologies that become available and adapted for them and also shape older adults’ self-perceptions of their digital capabilities [12,24,65]. It is critical to challenge stereotypes of older migrants as nonusers and passive users of ICTs to ensure that these technologies continue to adapt while considering older adults as a key user demographic.

Family is a central source of support for older adults’ adoption and navigation of new technologies [21,41], which mirrors the findings of this study. However, participants wanted to improve their digital competence to navigate ICTs independently and were engaged in the learning sessions. Challenges with troubleshooting their devices were a cause of frustration, and some participants reported an overreliance on family to facilitate technology use, which was not always effective in improving their digital knowledge and skills. Other studies have shown the importance of social support from family and friends in facilitating older adults’ access to and use of technologies [12,24], and this holds true for immigrant and refugee older adults. Family members of some immigrant and refugee older adults may also have limited digital competence, or immigrant and refugee older adults might be socially isolated without family support. Offering digital learning opportunities to increase their competence is a way to enhance digital agency [24]. Older adults might use technologies for necessity or leisure, so developing safe and engaging learning environments that meet their preferred needs is critical [12,21,22].

This study expands on existing literature on older migrants and ICT use [38,62,66] by exploring the digital competence of Arabic-speaking immigrant and refugee older adults who creatively circumvent challenges as they navigate the digital divide [24]. Migration shapes motives and needs for ICT adoption, with particular challenges arising for older migrants. Older migrants desire to stay connected with those left behind in their countries of origin, and they need to access local sources of support in their new country. ICTs will continue to play a vital role in communication patterns and information sharing for this population. Effective digital learning programs must be designed to overcome the intersecting barriers of age-related disabilities, cultural differences, language barriers, and low literacies. Canada has digital competence programs that target underserved older adults, such as the Digital Literacy Exchange Program [67] and the SUCCESS Digital Literacy Exchange Program for newcomers [68]. Participants in this study did not report accessing digital competence programs outside of the research study. While agism is often associated with the digital divide [24,59,65], digital exclusion based on language and language minority status must also be addressed when considering the opportunities available to older adults. Digital learning programs that integrate elements of English-as-an-additional-language learning, focus on problem-solving and creative uses of technologies, and tailor learning outcomes to the pragmatic needs of older migrants for transnational communication and local integration are likely to be the most successful. Finally, a discussion of ICT design is beyond the scope of this paper, but digital technologies that are acceptable to older adults and meet their needs, including for older adults with language and literacy barriers, are much needed as this demographic continues to grow worldwide.

### Strengths and Limitations

This study allowed for an in-depth exploration over a short period of Arabic-speaking immigrant and refugee older adults’ digital competence. A longitudinal design over a longer period could add further insights into evolving digital competencies in migrant older adults. Most study participants (20/31, 65%) were women, and hence, we were unable to explore gender differences. Some exceptions in this study related to women who reported constrained communication choices within transnational families and the importance of privacy in social media interactions. Other studies have shown how gender shapes the use of and access to technologies [40,69-71], and this is an area for further exploration. Most participants were healthy and younger older adults. Digital technology use and health are linked [12,72]; hence, further exploration of technology use in frail older migrants is required to meet their needs for social connectivity and information access. With the central role of social support and family in ICT adoption, future studies that include the perspectives of family members and caregivers might be critical in designing digital interventions for this population. Finally, the focus on Arabic-speaking older adults means that other populations of migrants might have differing experiences that warrant investigation.

### Conclusions

Canada’s immigrant and refugee older adult population will continue to grow, and addressing equitable access to ICTs is imperative to enhance quality of life in older age. Strengthening agency to use ICTs has the potential to markedly improve their social connectedness and access to local information. Digital competence education tailored to the needs of older migrants can empower active participation in the digital world and meet settlement needs after migration. These programs would need to focus on the competencies of communication and information access with attention to language preferences, transnational ICT-mediated practices, different types of literacies (eg, basic and informational), and creative troubleshooting strategies. Further research is needed to inform policy on innovative strategies for disseminating local information and resources to older migrants, such as leveraging social media platforms as well as developing ICTs that meet the needs of older adults more generally.

## Appendix

Multimedia Appendix 1 Topical codes—digital competence of participants.

## Abbreviations

DigComp: Digital Competence Framework for Citizens
FG: focus group
ICT: information and communications technology

## Data Availability

The datasets generated or analyzed during this study are not publicly available, due the nature of qualitative data where migrant research participants might be easily identifiable, but are available from the corresponding author on reasonable request.
