# Supplementary material for: Digital Competence of Arabic-Speaking Immigrant and Refugee Older Adults Enacting Agency and Navigating Barriers: Qualitative Descriptive Study
Source: J Med Internet Res. 2025 Mar 25;27:e60547. doi: 10.2196/60547 (PMC11979532; doi:10.2196/60547)
Supplement: Multimedia Appendix 1 [file jmir_v27i1e60547_app1.docx]

**Table S1.** Topical codes—digital competence of participants.^a^

|  | | Participant quotes |  |
| --- | --- | --- | --- |
| **Information and data literacy** | | |  |
|  | Browsing, searching, and filtering data, information, and digital content | - “You could navigate for information in Arabic, but you can’t be sure if what you found is a trusted site. Likewise, if you look it up in English, you can try to translate the information online into Arabic but it won’t be as easy to understand as if it were in Arabic to begin with.” [P6 and P7; knowledge] - “I do not know English, so I do not access government sites...In Canada, in the Province of Alberta, anything new on Facebook I receive information from (name), a Syrian activist who speaks Arabic and writes in Arabic, I take news about the province from his page. I see what is new in the Province.” [P8; FG^b^; skills] - “Recently P1 and 2 moved from where they were living before and are excited to be connected to the mosque newsletter. P3 already receives the mosque newsletter but wants to see if there is an Arabic version.” [Observation; attitudes] |  |
|  | Evaluating data, information, and digital content | - “Sometimes I see pictures that are one, two, or three years old and they write that this has happened today, but it’s obvious or I remember that it’s from a few years ago.” [P5; knowledge] - “Nowadays, to get the right information, sometimes I have to check more than one source and I compare the information...I exchange information with my friends and see if it’s accurate. See what they’ve heard, so they tell me just to try to get the most accurate information...some (friends) are here, and some are back home. It depends. Some in Egypt, some in a different country.” [P12; skills] - “We don’t trust everything on Facebook, I mean I don’t even trust things on Facebook, not even 10%, but I believe language resources are trustworthy, sports, games, sports game results, yeah, but I don’t trust any political news.” [P1; attitudes] |  |
|  | Managing data, information, and digital content | - “I don’t know how to (retrieve and save information that I have accessed)...I don’t know where to save it.” [P2; FG; knowledge] - “My way is that when I find information and it might be of interest to another person, I send it to them on WhatsApp and this way it is saved as I can see it on my end.” [P7; FG; skills] |  |
| **Communication and collaboration** | | |  |
|  | Interacting through digital technologies | - “P4 didn’t realize her friend requests were her friends and family as she can’t read in Arabic, so she is now adding them.” [Observation; knowledge] - “P1 after seeing this slide has mentioned that she gets email messages and she doesn’t understand, she doesn’t open the message and it is left unread. Doesn’t know how to respond to voicemail.” [Observation; skills] - “We learned that P4 isn’t using Facebook because she used to have it and accidentally deleted it and didn’t want to get it back or go through the trouble of re-downloading it.” [Observation; attitudes] |  |
|  | Sharing through digital technologies | - “Setting up friends who can see posts except for select contacts. This appears to be something they are all learning for the first time.” [Observation; knowledge] - “Going over Facebook slides. P4 wants to learn more about it...She knows how to add people and search for content. Doesn’t know how to block people on Facebook. P3 wants to learn how to share on Facebook.” [Observation; skills] - “Sometimes there are things like the skype and internet-related things like video calls when I talk with my kids, my wife helps me with that. I am not very good with technology because we’re a bit old school.” [P10; attitudes] |  |
|  | Engaging in citizenship through digital technologies | - “If I knew English, I would have followed Canadian groups on Facebook, of course but I don’t know English so I have to follow the Arabic groups.” [P1; knowledge] - “No, so they don’t know anything about accessing health information or banking information online, or any essential services, my understanding.” [P1, P2, and P3; group interview; skills] - “My knowledge is not that wide open to it. But thanks God, my grandkids taught me how to get my bank account and to see, you know, what’s in it, and to pay my bills.” [P9; attitudes] |  |
|  | Collaborating through digital technologies | - “Quran lessons on ZOOM.” [P6] - “I am on ZOOM, we all read the Quran on ZOOM.” [P3; FG; skills] |  |
|  | Netiquette | - “P3 wants to change the Facebook workshop group she is in, and this requires learning how to change her personal information on her Facebook account.” [Observation; knowledge] - “P6 wants to learn about how to block somebody on Facebook.” [Observation; skills] - “P5 has a large friend request list containing her in-law side of the family. Although P5 knows them, she didn’t want to add them as her friends on Facebook. She wants to keep her privacy, and noted that her husband has a large family (largest family in town), and she doesn’t want them on her Facebook.” [Observation; attitudes] |  |
|  | Managing digital identity | - “P3 wants to change the Facebook workshop group she is in, and this requires learning how to change her personal information on her Facebook account.” [Observation; skills] |  |
| **Safety** | | | |
|  | Protecting devices | - “Their bewilderment and lack of awareness says a lot—perhaps they’re learning that their children have set up a lot of the functions/accounts on their phones for them and handed the set-up phone to them after so the participants never needed to deal with the set-up process and account/password management.” [Observation; knowledge] - “P3 had a question about passwords and asked the instructor directly. He asked about what to do when choosing password variations (i.e. using symbols)...It turns out he had trouble finding where to get the symbol part of the keyboard, to which the instructor was able to show him on his phone keyboard.” [Observation; skills] - “Some participants didn’t care for privacy e.g. P7 and P6. P7 had mentioned something along the lines of ‘I’m old now, I have nothing to hide.’” [Observation; attitudes] |  |
|  | Protecting personal data and privacy | - “P4 asking about if google is safe. Worried about hacking and how pictures without the hijab would be stored. Instructor needed to explain how privacy works on iCloud and Google Photos storage which are more secure than on social media.” [Observation; knowledge] - “The participants who privatized their settings was P1, P2, P3, P4, P6. P8 as well though she already had private settings on.” [Observation; skills] - “I myself used to teach them how to deal with the scammers...If they call you, you got a call from an unknown number, an unknown person speaking English, ‘You understand English?’ Just say, ‘No English,’ hang up, that’s it.” [P11; attitudes] |  |
|  | Protecting health and well-being | - “Block and report someone on WhatsApp. Practicing how to block and unblock...P5 is showing and teaching P3 on her phone in Arabic.” [Observation; skills] - “P5 shared that ever since she moved to Canada she has family and friends asking for money so she wants to block them.” [Observation; attitudes] |  |
| **Problem-solving** | | |  |
|  | Solving technical problems | - “Participant 1 worked through deleting an email and restoring it with the instructor teaching him. He has never deleted an email before.” [Observation; knowledge] - “P3 wants to change the Facebook workshop group she is in, and this requires learning how to change her personal information on her Facebook account.” [Observation; skills] - “Meanwhile, P1 and P2 want to cancel voicemail because it is annoying and has no use. They don’t know how to use it. The instructor explained that when they miss a call (for example from the doctor or the government), a voicemail is left, and P1 and P2 said they were unaware of this. P1 and P2 mentioned that sometimes they get voicemails even when people don’t call them.” [Observation; attitudes] |  |
|  | Identifying needs and technological responses | - “The instructor and P4 were chatting, and he expressed that he doesn’t fully know the importance of email...He is learning that if he wants, he can open emails on a computer and not just his phone, he appears bewildered that it’s possible to do that. However, his son has the password to the email, so he is learning that he needs the password in order to access emails online.” [Observation; knowledge] - Skills:   - P1: “Also learning the English language, we downloaded apps.”   - P3: “We downloaded more than one app.”   - P1: “Here it is.”   - Translator: “Are there apps on Facebook that teach English?”   - P2: “Yes, yes.” - “Of course, not a lot of people want to go back and learn at this age and start all over again. I feel like my brain doesn’t accept it. For example, when my daughter tells me let’s create an email for you, I tell her to just do it and leave me out of it [laughter]. Older people don’t like this, I am not very motivated to learn, I feel like I’m good the way I am. Like, on the computer, maybe it’s not good for my eyes or my body to sit for a long time. Maybe I’ll get tired. I don’t know, maybe it’s a thing for all seniors, they like to run away from all this new technology.” [P13; attitudes] |  |
|  | Creatively using digital technologies | - Not identified |  |
|  | Identifying digital competence gaps | - “I don’t have any idea about anything...I don’t have somebody live with me, so when I stuck, I get so upset so that’s why I never try again.” [P7; FG; knowledge] - “I want to be able to learn how to use my phone. Like anytime I need anything, I have to ask for help: ‘can you help me with this, can you set up email for me, I don’t know how to do it.’ Same thing for the computer. I should know how to use it...it’s a necessity of life.” [P9; attitudes] |  |

^a^Digital content creation did not emerge as a priority area in discussions with participants, and it was beyond the scope of the project to address it in the learning sessions.

^b^FG: focus group.
